# Relationship between auxiliary gamma subunits and mallotoxin on BK channel modulation

**DOI:** 10.1038/srep42240

**Published:** 2017-02-06

**Authors:** Xin Guan, Qin Li, Jiusheng Yan

**Affiliations:** 1 Department of Anesthesiology and Perioperative Medicine, The University of Texas MD Anderson Cancer Center, Houston, Texas, USA

## Abstract

The large-conductance, calcium- and voltage-activated K^+^(BK) channel consists of the pore-forming α subunits (BKα) and auxiliary subunits. The auxiliary γ1-3 subunits potently modulate the BK channel by shifting its voltage-dependence of channel activation toward the hyperpolarizing direction by approximately 145 mV (γ1), 100 mV (γ2), and 50 mV (γ3). Mallotoxin is a potent small-molecule BK channel activator. We analyzed the relationship between mallotoxin and the γ subunits in their BK channel-activating effects in membrane patches excised from HEK-293 cells. We found that mallotoxin, when applied extracellularly, shifted the half-activation voltage (V_1/2_) of BKα channels by −72 mV. The channel-activating effect of mallotoxin was greatly attenuated in the presence of the γ1, γ2, or γ3 subunit, with resultant ΔV_1/2 (+/− mallotoxin)_ values of −9, −28, or −15 mV, respectively. Most examined γ1 mutant subunits antagonized mallotoxin’s channel-activating effect in a manner that was largely dependent on its own modulatory function. However, mallotoxin caused an irreversible functional and structural disengagement of the γ1-F273S mutant from BK channels. We infer that the auxiliary γ subunit effectively interferes with mallotoxin on BK channel modulation via either a direct steric competition or an indirect allosteric influence on mallotoxin’s binding and action on BKα.

The mammalian big/large-conductance, voltage- and calcium-activated potassium (BK) channel is widely expressed in various tissues and cell types and plays important roles in many physiological processes, including contractile activity of smooth muscles^1^, regulation of neurotransmitter release and neuronal firing^2^,^3^,^4^,^5^, and frequency tuning of auditory hair cells^6^. The BK channel features large single-channel conductance and dual activation by membrane depolarization and elevation of intracellular free calcium ([Ca^2+^]_i_). BK channels consist of the pore-forming, voltage- and Ca^2+^-sensing α subunits (BKα), either alone or together with tissue-specific auxiliary β subunits (β1–β4) or γ subunits (γ1–γ4)^7^,^8^,^9^. The BK channel γ subunits (BKγ) exhibit different tissue-specific mRNA expression and may broadly modulate BK channels in different tissues^9^. The few published studies have shown that the γ1 subunit regulates BK channels in prostate cancer cells^7^,^8^, salivary gland cells^10^, airway epithelial cells^11^,^12^, and also probably in arterial smooth muscle cells^13^.

The auxiliary γ subunits are a group of four leucine-rich repeat (LRR)–containing membrane proteins, γ1 (LRRC26), γ2 (LRRC52), γ3 (LRRC55), and γ4 (LRRC38). They are structurally distinct from the double membrane–spanning BK channel β subunits in possessing an N-terminal signal peptide, an extracellular LRR domain, a single transmembrane segment, and a short intracellular C-terminus^8^,^9^,^14^. The γ1, γ2 and γ3 subunits, all ~35 kDa in size, facilitate BK channel activation by shifting voltage-dependence of channel activation in the hyperpolarizing direction over an exceptionally large range, by approximately 145 mV (γ1), 100 mV (γ2), and 50 mV (γ3) in the absence of calcium^8^,^15^.

In addition to their regulation by auxiliary proteins, BK channels are modulated by a variety of endogenous or exogenous small peptide or chemical molecules^16^,^17^,^18^,^19^. The pharmacological properties of BK channels are extensively altered in the presence of the auxiliary β subunits^20^. The interactions between the β subunits and small molecule modulators of BK channels have been a subject of intensive investigations^20^,^21^. However, currently little is known about the influence of the newly identified γ subunits on the BK channel’s pharmacological properties.

Mallotoxin, also called rottlerin, is the principal phloroglucinol constituent of kamala, a red powder on the surface of the fruit of the kamala tree (*Mallotus philippinensis*) that has been used as a folk medicine in treating tapeworm infection, eye diseases, bronchitis, abdominal disease, spleen enlargement, and other illnesses^22^. At micromolar concentrations, mallotoxin potently shifted the voltage-dependence of BK channel activation toward the hyperpolarizing potential direction when applied extracellularly on whole cells^23^. The BK channel–activating effect of mallotoxin was largely abolished when the γ1 subunit was either endogenously expressed in salivary gland cells or heterologously expressed in human embryonic kidney (HEK)-293 cells^10^. Despite the potent activating effects of the γ1 subunit and mallotoxin on BK channels, the mechanisms underlying their individual actions and their interactions within BK channel complexes are largely unknown. In this study, we analyzed the relationships between the BK channel auxiliary γ subunits and mallotoxin and probed the mechanisms underlying the apparent antagonizing effect of the γ subunits on mallotoxin’s channel-activating function.

## Results

### Effects of mallotoxin on BKα channels in excised membrane patches

**Figure 1:**
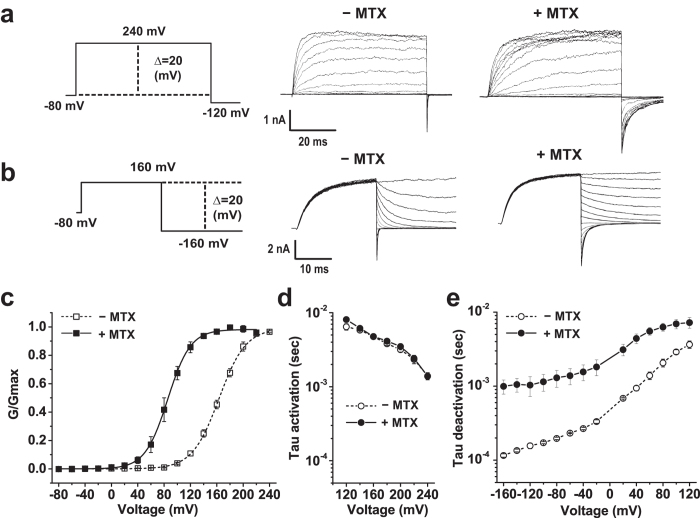
Effects of mallotoxin on BK channels formed by BKα alone. (**a,b**) Voltage protocols (left) and the representative activation (**a**) and deactivation (**b**) currents of the BKα channel at different voltages in the absence (−MTX) and presence of mallotoxin (+MTX). (**c)** Voltage-dependence of BK channel activation. (**d,e**) Voltage-dependence of the kinetics of channel activation (**d**) and deactivation (**e**).

We observed, in excised patches of HEK-293 cell membrane, that extracellular exposure of the heterologously expressed BKα channels to mallotoxin potently shifted the voltage-dependence of channel activation. Mallotoxin was included in pipette solution under an inside-out configuration and data was collected after the mallotoxin achieved its maximal effect in shifting the BK channel’s conductance-voltage (G-V) relationship towards hyperpolarizing direction. The results from mallotoxin treatment were compared to those without mallotoxin that were obtained in separate patches. Mallotoxin at 2 μM resulted in a large shift of 72 mV in the half-activation voltages (V_1/2_) of BK channels toward the hyperpolarizing direction (to 92 ± 6 mV, n = 4), compared to the untreated BKα channels (V_1/2_ = 164 ± 3 mV, n = 11) in the virtual absence of [Ca^2+^]_i_ (Fig. 1a and c; Table 1). The mallotoxin-induced left shift in the conductance-voltage (G-V) relationship was accompanied by a great deceleration in channel deactivation (e.g., ~10 times decrease in rate at −160 mV), while the rate of channel activation at tested voltages (120–200 mV) was largely unaffected (Fig. 1b,d and e). Increase in mallotoxin concentration from 2 to 10 and 20 μM showed no significant increase in modulatory effect on BK channels. We also observed that mallotoxin at a high concentration, e.g., 10 or 20 μM, precipitated in pipette solution. Thus, 2 μM mallotoxin was used throughout this study except where another concentration is specified.

### Effects of mallotoxin on BK channels in the presence of auxiliary γ subunits

**Figure 2:**
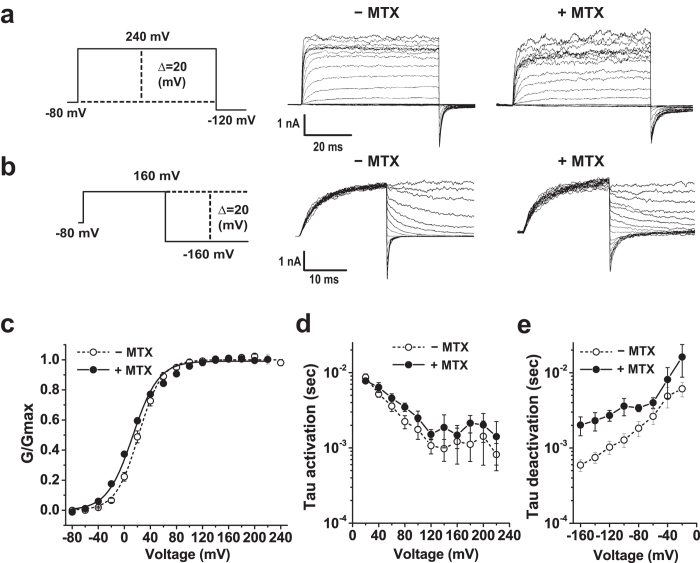
Effects of mallotoxin on BK channels in the presence of the auxiliary γ1 subunit. (**a,b**) Voltage protocols (left) and the representative activation (**a**) and deactivation (**b**) currents of BKαγ1 channel complexes at different voltages in the absence (−MTX) and presence of mallotoxin (+MTX). (**c**) Voltage-dependence of BK channel activation. (**d,e**) Voltage-dependence of the kinetics of channel activation (**d**) and deactivation (**e**).

To test whether the auxiliary γ subunits affect mallotoxin’s channel-activating effect on BK channels, we co-expressed γ1, γ2, γ3, and γ4 individually with BKα in HEK-293 cells. In the presence of the γ1 subunit, application of mallotoxin caused no significant change in BK channel V_1/2_ (ΔV_1/2_ = −9 mV). The V_1/2_ values of mallotoxin-treated and -untreated BKαγ1 channels in the virtual absence of [Ca^2+^] were 14 ± 2 mV (n = 5, fitted by single Boltzmann function) and 23 ± 3 mV (n = 10), respectively (Fig. 2a,c; Table 1). The BKαγ1 channel deactivation was moderately slower in the presence of mallotoxin (e.g., ~3 times slower at −160 mV) than in the absence of mallotoxin, while the channel activation was less affected (Fig. 2b,d and e). Application of mallotoxin on the membrane patches over an extended time (e.g., more than 15 minutes) had little further effect on the G-V relationship, but the membrane patches appeared to be less stable and more leaky, presumably due to some non-specific effects of the hydrophobic mallotoxin on cell membranes. Therefore, all electrophysiological data presented in this study were collected within 5–10 minutes of mallotoxin application.

**Figure 3:**
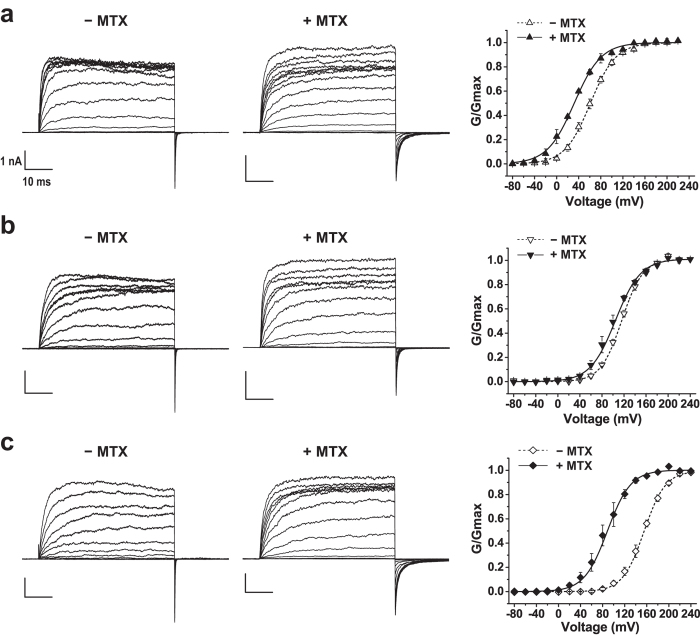
Effects of mallotoxin (MTX) on voltage-dependence of BK channel activation in the presence of the auxiliary subunits γ2 (**a**), γ3 (**b**), or γ4 (**c**). The voltage protocol used for data recording is the same as in Figs 1a and 2a. Reprehensive activation currents of the BKαγ1-3 channels are in the absence and presence of mallotoxin and the plotted voltage-dependence of BK channel activation are shown on the left, middle, and right sides, respectively.

The γ2 subunit also exerted a significant attenuating effect on mallotoxin. Mallotoxin produced a shift of only −28 mV in BK channel voltage-dependence of activation, from 61 ± 3 mV (n = 8) for the untreated BKαγ2 channel to 33 ± 2 mV (n = 3) for the treated channels (Fig. 3a; Table 1). The presence of the γ3 subunit, like the γ1 subunit, abolished most of the channel-activating effect of mallotoxin, with a difference of only −15 mV in the BKαγ3 channel V_1/2_ values in the presence (100 ± 5 mV, n = 4) and absence (115 ± 2 mV, n = 6) of mallotoxin (Fig. 3b; Table 1). The γ4 subunit, which was barely effective in BK modulation, had no significant effect on mallotoxin’s channel-activating effect. Mallotoxin induced a significant shift of −69 mV in BK channel V_1/2_, from 154 ± 3 mV (n = 7) for the untreated BKαγ4 channels to 85 ± 8 mV (n = 4) for the treated channels (Fig. 3c; Table 1), a result similar to that with BKα channels in the absence of γ subunits.

### Inverse correlation of the activating effects of mallotoxin on BK channels and the modulatory function of most BKγ1 mutants

**Figure 4:**
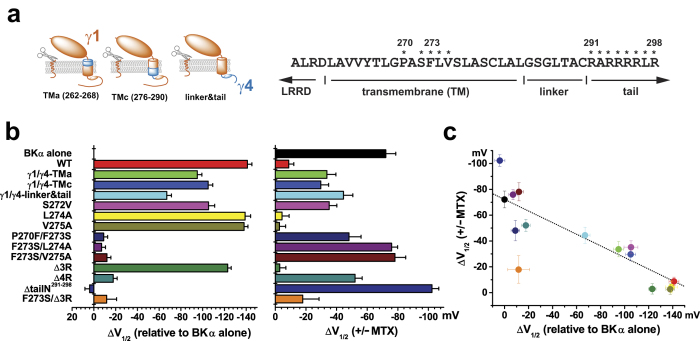
The relationship between the γ1 subunit and mallotoxin (MTX) in determining their efficacies in shifting the BK channel V_1/2_. (**a**) The schematic structures of the used chimeric γ1/γ4 mutants and the location of mutations in amino acid sequence. (**b**) The shifts in BK channel V_1/2_ (ΔV_1/2_) induced by wild-type (WT) and mutant γ1 subunits in the absence of mallotoxin (left) and by mallotoxin in the presence of the corresponding γ1 proteins (right). (**c**) The relationship between the values of ΔV_1/2_ induced by γ1 and mallotoxin. For reference, a straight line was drawn between the two data points of BK channels in the absence of γ1 and in the presence of γ1-WT.

Our group recently reported that a peptide region of the γ subunit encompassing the single transmembrane segment and its neighboring intracellular polybasic charged cluster are responsible for the difference in modulatory functions of the four γ subunits^24^ and also are the key determinants for the γ1 subunit’s modulatory function^25^. To define the relationship between the γ1 subunit’s modulatory function and the channel-activating effect of mallotoxin, we analyzed the modulatory effects of mallotoxin in the presence of various γ1 mutants, which displayed a range of different capacities for shifting the BK channel V_1/2_ toward the hyperpolarizing direction via mutations in the transmembrane or C-tail region^25^. It is notable that, for most of the 13 γ1 mutants examined, their V_1/2_-shifting capacities were inversely correlated with the mallotoxin’s V_1/2_-shifting effect in the mutant’s presence (Fig. 4; Table 1). The BK channel-activating effect of mallotoxin was not significantly attenuated by most of the loss-of-function mutants. The F273S/P270V, F273S/L274A, F273S/V275A, Δ4 R, and ΔtailN^291-298^ mutants lost ≥87% of the V_1/2_-shifting capacity of the wild-type (WT) γ1 subunit. Consequently, they were largely unable to antagonize the mallotoxin-induced shift in BK channel V_1/2_ (ΔV_1/2_ = −52 to −102 mV). The L274A, V275A, and Δ3 R mutants retained more than 85% of the V_1/2_-shifting capacity of the γ1-WT subunit. Like the γ1-WT subunit, they also largely suppressed the BK channel–activating effect of mallotoxin, with nearly no noticeable shift in V_1/2_ (|ΔV_1/2_| ≤5 mV) between the treated and untreated membrane patches. The four partially functional mutants, γ1/γ4-linker&tail, γ1/γ4-TMa, γ1/γ4-TMc, and S272V, shifted BK channel V_1/2_ in a range of −67 to −100 mV (48–75% of the capacity of the γ1-WT subunit) in the absence of mallotoxin. Their presence only partially antagonized the BK channel–activating effect of mallotoxin, and the resultant ΔV_1/2 (+/− mallotoxin)_ values were −44 mV, −34 mV, −30 mV, and −40 mV, respectively (Fig. 4b; Table 1), which are close to a line drawn between the effects of mallotoxin on the BKα channels and on the BKαγ1^WT^ channels (Fig. 4c; Table 1). These results showed that the γ1 subunit antagonized the mallotoxin’s channel-activating effect in a manner that is largely dependent on its own modulatory function. However, the F273S/Δ3 R mutant appeared to be exceptional which was inactive in BK channel modulation but still effective in attenuating the BK channel–activating effect of mallotoxin, with a resultant ΔV_1/2 (+/− mallotoxin)_ value of only 29 mV.

### Mallotoxin irreversibly disengaged the γ1-F273S mutant from BK channels

**Figure 5:**
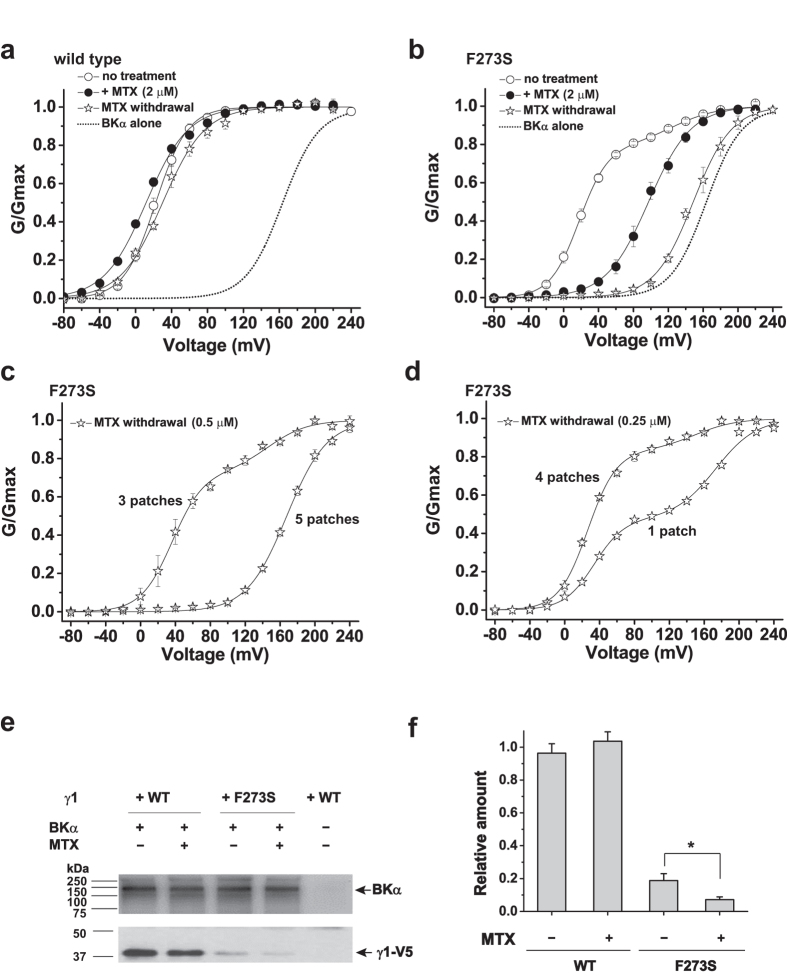
Mallotoxin disengaged γ1-F273S mutant from BK channel modulation. (**a**) Mallotoxin (MTX) at 2 μM had little effect on the G-V relationship of BK channels formed by BKα and the wild-type γ1 subunit (γ1-WT) during treatment and after withdrawal of mallotoxin. (**b**) Mallotoxin at 2 μM induced an irreversible drastic loss of the channel-modulatory function of the γ1-F273S mutant. (**c**,**d**) Treatment of cells with a low concentration of mallotoxin produced less loss of the channel-modulatory function of the γ1-F273S mutant. A double Boltzmann function was used to generate G-V fitting curves for the data of no mallotoxin treatment in (**b**) and of mallotoxin withdrawal for 3 patches in (**c**) and for all patches in (**d**). (**e**) Immunoblot analysis of the BKαγ1 channel complex immunoprecipitated with anti-BKα antibody in the absence or presence of mallotoxin. BKα and γ1-V5 were immunoblotted with anti-BKα and anti-V5 antibody, respectively. (**f**) Densitometric analysis of the relative content of γ1-V5. Data were collected from six independent experiments. For each experiment, the intensity of the γ1-V5 band was first normalized to that of the BKα band and then to the mean of untreated and treated WT samples. *p < 0.05.

We recently reported that the F273S mutant of the γ1 subunit (γ1-F273S) resulted in weakened association between the γ1 and BKα subunits, which caused a small fraction (~20%) of the channels to be high voltage–activated channels with a V_1/2 _≥ 100 mV^25^. We found that the total expression of F273S was not significant different from WT but overexpression of the F273S mutant enhanced its modulatory function by increasing the portion of low V_1/2_ channel from 79 to 91%, suggesting a likely deceased binding affinity of the F273S mutant to BKα^25^. Interestingly, in contrast to the slightly activating effect of mallotoxin on the BK channels in the presence of γ1-WT (Fig. 5a; Table 1), mallotoxin exerted a significant inhibitory effect on BK channels initially complexed with γ1-F273S by shifting the G-V relationship toward to the positive voltage direction with a V_1/2_ value of 96 ± 5 mV which is close to that of the mallotoxin-modulated BKα alone channels (Fig. 5b; Table 1). We further found that the modulatory function of the F273S mutant was, indeed, largely irreversibly lost upon pre-application (5 min) and then withdrawal of mallotoxin, with a resultant V_1/2_ of 147 ± 3 mV, which is close to that of the BKα channel alone (Fig. 5a; Table 1). In contrast, there was no drastic difference for the BK channel’s G-V relationship before mallotoxin treatment and after mallotoxin withdrawal in the presence of γ1-WT (Fig. 5a; Table 1). As expected from a competition of mallotoxin with γ1-F273S for BK channel modulation, we found that a reduction in the concentration of mallotoxin to 0.5 or 0.25 μM during the pre-treatment resulted in a great decease in the loss of the channel modulatory function of γ1-F273S. After mallotoxin withdrawal, the channel modulatory effect of the γ1-F273S mutant was largely retained in 3 out of 8 patches for cells pretreated with 0.5 μM mallotoxin (Fig. 5c) and fully retained in 4 patches and only partially lost in 1 patch (totally 5 patches) for cells pretreated with 0.25 μM mallotoxin (Fig. 5d).

To determine whether mallotoxin can affect the physical association between the γ1 and BKα subunits, we performed immunoprecipitation and immunoblot analyses of the BKαγ1^WT^ and BKαγ1^F273S^ channel complexes in the absence and presence of mallotoxin (Fig. 5e and f). The BK channel complex was immunoprecipitated by immobilized anti-BKα antibody that was covalently crosslinked to the agarose beads and then immunoblotted with anti-BKα and anti-V5 antibodies for the BKα subunit and the V5-tagged γ1 subunit, respectively. Similar to the recently reported weakened association between the γ1-F273S mutant and BKα^25^, only ~20% of the γ1-F273S mutant protein, as compared to γ1-WT, remained associated with BKα in the isolated channel complexes. Mallotoxin treatment caused a further >60% reduction in the BKα-associated γ1-F273S mutant protein in the isolated channel complexes. However, no significant difference in the association of the γ1-WT subunit to BKα was observed in the isolated channel complexes in the absence or presence of mallotoxin treatment.

## Discussion

In this study, we found that mallotoxin, when applied extracellularly on excised membrane patches, shifted the half-activation voltage (V_1/2_) of BK channels by −72 mV in the absence of γ subunits. The channel-activating effect of mallotoxin was largely abolished or greatly attenuated in the presence of subunits γ1, γ2, and γ3, with resultant ΔV_1/2 (+/− mallotoxin)_ values of −9, −28, and −15 mV, respectively. Most of the examined γ1 mutant subunits antagonized mallotoxin’s channel-activating effect in a manner that was largely dependent on its own modulatory function. However, mallotoxin caused an irreversible functional and structural disengagement of the γ1-F273S mutant from BK channels.

Mallotoxin has been found to affect numerous cellular events, including ion channel activation^23^,^26^, many protein kinase signaling pathways^27^,^28^, induction of mitochondrial uncoupling^29^, and autophagy^30^. The previous electrophysiological studies on the effects of mallotoxin on BK channels were mostly recorded in whole cell configuration^10^,^23^, which could be complicated by mallotoxin’s effect on intracellular events, such as many kinase activities^27^,^28^. The present study relied on patch-clamp recording on excised membrane patches to minimize interference from mallotoxin-induced intracellular events. The observed ~70 mV shift in the voltage-dependence of BKα channel gating in HEK-293 cells in the presence of 2 μM mallotoxin is close to that observed in whole cell recording in CHO-K1 cells (70 mV at 5 μM)^10^ but smaller than that originally reported through whole cell recording in HEK-293 cells (>100 mV at 0.5 μM)^23^. The discrepancy could be due to a difference in cell recording configurations. We also confirmed that, in excised membrane patches, the γ1 subunit largely attenuated the BK channel-activating effect of mallotoxin (ΔV_1/2_ = 9 mV at 2 μM), a result similar to that previously observed in whole cell recording of native channels in parotid acinar cells (ΔV_1/2_ = 6 mV at 5 μM) and recombinant BKα-γ1 channels in CHO-K1 cells (ΔV_1/2_ = 17 mV at 5 μM)^10^. Our observation that the γ2 and γ3 subunits also largely attenuated the BK channel–activating effect of mallotoxin suggests that the pharmacological properties of BK channels exerted by the three different auxiliary γ subunits are similar. Therefore, the results we obtained on excised membrane patches confirmed previous findings on whole cell recording^10^,^23^ and further demonstrated that mallotoxin directly activated BK channels in the absence of the auxiliary γ subunit but became largely ineffective in the presence of the auxiliary γ1, γ2, and γ3 subunits.

To probe the mechanism underlying the drastic difference in the activating efficacy of mallotoxin on the BKα and BKαγ1 channels, we examined the effects of mallotoxin on BK channels complexed with various γ1 mutants. Our finding that, for most γ1 subunit mutants tested, the BK channel V_1/2_-shifting capacity was correlated with their antagonizing effects on the mallotoxin’s channel-activing function. One may argue that channel-activating function of mallotoxin is simply dependent on the extent of channel-stimulation or -activation by any other activator through an uncompetitive mechanism which involves two independent binding/active sites. For example, the γ1 subunit might affect the efficacy of mallotoxin by maximally affecting the same gating parameter, e.g., the allosteric coupling factor between the voltage sensors and channel pore gate^8^,^10^. However, an uncompetitive mechanism can be ruled out by three lines of evidence. First, the effect of mallotoxin on the BKα channel was largely unaffected by another activator, the ligand Ca^2+^ 23. Second, the γ3 subunit and the γ1-F273S/Δ3 R mutant, which are a much weaker modulator than γ1-WT or a nearly loss-of-function modulator, respectively, were still effective in suppressing mallotoxin’s channel-activating effect. Third, as discussed next, mallotoxin can structurally compete off the γ1-F273S mutant from BK channels.

We observed that the γ1 subunit and mallotoxin mutually affected each other on their modulatory effects on BK channels. The mallotoxin-induced shifts in the G-V curves toward the depolarizing direction in the initial presence of the F273S mutant can be explained by mallotoxin-induced displacement of the F273S mutant protein from the BK channels, evidenced by the irreversible loss of BK channel modulation by the F273S mutant upon withdrawal of mallotoxin and also the mallotoxin treatment–induced dissociation of the F273S mutant protein in the immunoprecipitated channel complexes. The F273 residue was predicted to be near or in the middle of the single transmembrane segment of the γ1 subunit and was found to play an important role in the γ1 subunit’s association with and modulatory function on BK channels^25^. Therefore, the γ1 subunit and mallotoxin can mutually affect their modulatory efficacy on BK channels in a specific γ1 transmembrane residue–dependent manner. This result can be explained by either a competitive or a noncompetitive mechanism between the γ subunit and mallotoxin for their mutual influence on BK channel modulation. For a competitive mechanism, the γ subunit and mallotoxin sterically compete on overlapping binding or active sites on BKα. The F273S mutation in the middle of the γ1 subunit’s transmembrane domain might create room and/or additional interaction to allow the mallotoxin to bind and thus to irreversibly disengage the γ1 subunit from BK channels. For a noncompetitive mechanism, the binding/active sites of the γ1 subunit and mallotoxin on BKα are distinct but allosterically and negatively coupled. The ability of mallotoxin to functionally and structurally disengage the γ1-F273S mutant could be simply due to the latter’s weakened association to BKα. For either of these two mechanisms, these γ1 mutants with partially reduced G-V shifting capacities likely exert less influence on the mallotoxin’s binding on BKα than the WT and thus allow mallotoxin to regain influence on BK channel gating. More studies will be needed to distinguish these two different mechanisms.

The mechanism for the decreased association of the F273S mutant to BKα remains unclear. We found that overexpression of the F273S mutant enhanced its modulatory function by increasing the portion of low V_1/2_ channel from 79 to 91%, suggesting the possibility of either a deceased expression or a comprised binding affinity to BKα.

## Materials and Methods

### Heterologous expression of BKα and γ proteins in HEK-293 cells

Recombinant cDNA constructs of human BKα (hSlo), γ1-4 subunits, and γ1 mutants were used for heterologous expression in HEK-293 cells. As described previously^8^,^9^,^24^, fusion cDNA constructs that encode precursor fusion proteins of human BKα and C-terminal–tagged BKγ proteins were generated with the pCDNA6 vector and used to facilitate the co-translational assembly of BKαγ protein complexes after endogenous cleavage by peptidases at the linker (signal peptide) region in the mature proteins. HEK-293 cells were obtained from ATCC. The cells were transfected with plasmids using Lipofectamine 2000 (Invitrogen) and subjected to electrophysiological assays 16–24 h after transfection.

### Electrophysiology

Inside-out BK channel currents were acquired at room temperature using EPC-10 (HEKA). Symmetric internal and external patch-clamp recording solutions contained 136 mM KMeSO_3_, 4 mM KCl, and 20 mM HEPES (pH = 7.20). The external solution was supplemented with 2 mM MgCl_2_, and the internal solution was supplemented with 5 mM HEDTA without Ca^2+^ to achieve virtual 0 [Ca^2+^]_i_. All patch-clamp data were collected at virtual 0 [Ca^2+^]_i_. Steady-state activation was expressed as the normalized conductance (G/Gmax) calculated from the relative amplitude of the tail currents (deactivation at −120 mV). The voltage of half-maximal activation (V_1/2_) and the equivalent gating charge (*z*) were obtained by fitting the relations of G/Gmax vs. voltage with the single Boltzmann function G/Gmax = 1/(1 + *e*^−*z*F(V-V1/2)/RT^) or with the double Boltzmann function G/Gmax = a/(1 + *e*^−*za*F(V-Va1/2)/RT^) + (1 − a)/(1 + *e*^−*zb*F(V-Vb1/2)/RT^). Mallotoxin was purchased from Sigma-Aldrich, stored at −20 °C in aliquots in DMSO, and freshly diluted and applied in the external recording solution at a final concentration of 2 μM. For mallotoxin withdrawal experiments, the cells were pretreated with 2 μM mallotoxin for 5 minutes in phosphate-buffered saline (PBS) buffer (pH 7.4) and then transferred to mallotoxin-free solution and subjected immediately to patch-clamp recording.

### Immunoprecipitation and immunoblotting

HEK-293 cells expressing the BKαγ1^WT^ or BKαγ1^F273S^ channel complexes were preincubated with 2 μM mallotoxin in PBS buffer (pH 7.4) for 5 minutes. The channel complexes were then solubilized from cell membranes with 1% dodecyl maltoside (DDM) in Tris-buffered saline (TBS) buffer (50 mM Tris, 150 mM NaCl, pH 7.6) supplemented with 2 μM mallotoxin. After centrifugation at 17,000 *g* for 10 minutes, the solubilized channel complexes in the supernatant were incubated for 2 hours with mouse monoclonal anti-BKα antibody (University of California–Davis/NIH Neuromab facility) that was covalently crosslinked to protein-A/G agarose beads (Thermo Fisher Scientific). The captured protein complexes were washed three times with TBS buffer supplemented with 1% DDM and 2 μM mallotoxin, eluted with SDS-PAGE sample buffer, and then loaded directly to 12% SDS-PAGE gel to be separated by electrophoresis. Resolved proteins were transferred to PVDF membranes (Thermo Fisher Scientific) and probed by a mouse monoclonal anti-V5 antibody (1:10000, Invitrogen) for V5-tagged γ subunits and a mouse monoclonal anti-BKα antibody (1:1000, University of California–Davis) for BKα. The intensities of the protein bands were analyzed with ImageJ software (US National Institutes of Health).

### Statistical analyses

Experimental values are reported as means ± standard error of the mean. The Student *t*-test was used for comparison of two groups. Differences were considered statistically significant at *p* < 0.05.

## Additional Information

**How to cite this article:** Guan, X. *et al*. Relationship between auxiliary gamma subunits and mallotoxin on BK channel modulation. *Sci. Rep.* **7**, 42240; doi: 10.1038/srep42240 (2017).

**Publisher's note:** Springer Nature remains neutral with regard to jurisdictional claims in published maps and institutional affiliations.

## Figures and Tables

**Table 1:** Boltzmann fit parameters of voltage-dependent BK channel activation.

Expression	No mallotoxin			+2 μM mallotoxin		
	V_1/2_ (mV)	*z*	*n*^a^	V_1/2_ (mV)	*z*	*n*
BKα alone	164 ± 3	1.28 ± 0.05	11	92 ± 6	1.32 ± 0.05	4
+γ1	23 ± 3	1.57 ± 0.11	10	14 ± 2	1.29 ± 0.10	6
+γ1*	27 ± 6	1.10 ± 0.06	4			
+γ2	61 ± 3	1.17 ± 0.07	8	33 ± 2	1.10 ± 0.12	3
+γ3	115 ± 2	1.36 ± 0.05	6	100 ± 5	1.13 ± 0.03	4
+γ4	154 ± 3	1.27 ± 0.07	7	85 ± 8	1.22 ± 0.09	4
+γ1/γ4-linker&tail	97 ± 3	1.03 ± 0.03	5	53 ± 6	1.00 ± 0.11	5
+γ1/γ4-TMa	69 ± 3	1.33 ± 0.05	3	35 ± 5	1.15 ± 0.15	4
+γ1/γ4-TMc	59 ± 3	1.11 ± 0.09	4	29 ± 4	1.19 ± 0.06	5
+γ1-S272V	64 ± 5	1.02 ± 0.05	5	24 ± 1	1.25 ± 0.15	3
+γ1-L274A	25 ± 4	1.43 ± 0.13	3	20 ± 2	1.35 ± 0.10	4
+γ1-V275A	26 ± 2	1.60 ± 0.13	3	23 ± 4	1.00 ± 0.18	3
+γ1-P270V/F273S	155 ± 2	1.48 ± 0.14	5	107 ± 8	0.98 ± 0.05	4
+γ1-F273S/L274A	157 ± 2	1.25 ± 0.19	3	81 ± 3	1.05 ± 0.06	5
+γ1-F273S/V275A	152 ± 2	1.14 ± 0.07	4	74 ± 7	0.76 ± 0.06	5
+γ1-Δ3 R	40 ± 1	1.34 ± 0.05	4	38 ± 4	1.07 ± 0.05	6
+γ1-Δ4 R	146 ± 4	1.15 ± 0.05	11	94 ± 4	0.93 ± 0.15	3
+γ1-F273S/Δ3 R	163 ± 7	1.13 ± 0.04	5	134 ± 6	1.00 ± 0.03	5
+γ1-ΔtailN^291-298^	168 ± 3	1.22 ± 0.11	5	66 ± 3	0.97 ± 0.07	4
+γ1-F273S	18 ± 1 (79%)^b^	1.55 ± 0.07^b^	13	96 ± 5	1.12 ± 0.05	5
	126 ± 8 (21%)^b^,^c^	0.99 ± 0.16^b^,^c^				
+γ1-F273S*	147 ± 3	1.14 ± 0.08	6			

^*^Condition of mallotoxin withdrawal in which cells were preincubated with 2 μM mallotoxin for 5 min and then transferred to a mallotoxin-free solution.

^a^*n* values are the number of recorded excised inside-out patches from different HEK-293 cells.

^b^The indicated percentage in parentheses here refers to the portion of the channels’ subpopulation that was obtained from a double Boltzmann function fit.

^c^Because of the difficulty in obtaining reliable parameter values from a double Boltzmann function fit for the minor portion (e.g., ≤35%), the estimated values of the V_1/2_ and errors provided here are considered less reliable and used for reference only.
